# Evaluating observed and perceived experiences of operating room to paediatric critical care unit handoffs: an initial assessment to inform quality improvement

**DOI:** 10.3389/fped.2025.1644064

**Published:** 2025-10-08

**Authors:** Jacob Davidson, Emily Walser, Evelyn V. Waugh, Claire A. Wilson, Julie Strychowsky, Janice Tijssen, Abhijit Biswas, Jennifer Y. Lam

**Affiliations:** ^1^ Division of Paediatric Surgery, Children’s Hospital, London Health Sciences Centre, London, Ontario, ON, Canada; ^2^ Department of Surgery, Schulich School of Medicine & Dentistry, Western University, London, Ontario, ON, Canada; ^3^ Department of Otolaryngology-Head & Neck Surgery, Schulich School of Medicine & Dentistry, Western University, London, Ontario, ON, Canada; ^4^ Department of Paediatrics, Schulich School of Medicine & Dentistry, Western University, London, Ontario, ON, Canada; ^5^ Department of Anesthesia and Perioperative Medicine, Schulich School of Medicine & Dentistry, Western University, London, Ontario, ON, Canada

**Keywords:** quality improvement, pediatric, handover, critical care, operating room, surgery

## Abstract

**Introduction:**

Transferring critically ill patients from the Operating Room (OR) to the Paediatric Critical Care Unit (PCCU) is a complex process. Unstructured handoffs and poor communication increase the risk of adverse events. This project aimed to characterize the current handoff process, identify strengths and deficiencies, and define opportunities for improving patient handover.

**Methods:**

A working group with multidisciplinary stakeholder representation was created. An audit tool was developed and used to evaluate daytime OR to PCCU handoffs. A survey was distributed electronically to all staff involved in the handoffs.

**Results:**

Audits of 50 handoffs revealed that only 71.4% of handoffs included the full perioperative team and introductions were rarely completed (14.0%). The majority (81.8%) of the Anaesthesia content was discussed consistently (>60% of the time). In contrast, over half (53.8%) of surgical elements were discussed less than 50% of the time. Sixty-two survey responses revealed team members were often absent (67.0%) or inattentive (45.0%), and handoffs lacked clarification and wrap-up (38.0%). Twenty-two percent of respondents felt information was missed and 60.0% were unsatisfied with the current handoff process. Siloed communication, need for standard pre-handoff information, and a structured handoff process were identified in survey comments.

**Conclusion:**

Audit and survey data identified multiple areas for process improvements in OR to PCCU handoffs. The combination of objective and subjective data enhanced results and informed future quality improvement efforts by engaging team members. These findings will aid in the development of a structured OR to PCCU handoff process to ensure effective and safe patient care.

## Introduction

A handoff (or handover) is defined as the transfer of information, professional responsibility, and accountability between individuals and teams (1). Handoffs occur on a routine basis for hospitalized patients, however, the transfer of a pediatric patient's care from the Operating Room (OR) to the Pediatric Critical Care Unit (PCCU) is a complex system that is susceptible to error if attention to detail is not taken. These critically ill patients are often medically complex, have multiple comorbidities, and/or have significant acute medical concerns. Additionally, patients returning from the OR who require ongoing critical care in the PCCU tend to have a number of physical lines, tubes, and drains that add complexity to the handoff process. Consequently, the transition of care between providers and provider teams from the OR to the PCCU is a known high-risk time point for errors to occur as a result of poor communication, missed information, or lack of structure in the handoff process (1–4).

The literature reports ineffective communication in general as a leading contributor to adverse events including medical errors and patient harm (5). The Joint Commission has identified communication as a root cause in up to 60% of sentinel events between 2012 and 2014 (6), while the Agency for Healthcare Research and Quality identifies through the Survey on Patient Safety Culture that information is lost in over half of handoffs performed (7). It is clear that efforts are needed to improve communication and teamwork and given the complex nature of the OR to PCCU handoff, specific care must be dedicated to this high-risk time point in an attempt to improve continuity of care and patient safety.

The use of a structured and standardized handoff process from the OR to the PCCU has been implemented and studied in a few Canadian centres and many more US institutions, to date (1–3, 8–11). These studies have shown a reduction in technical errors, omissions of information exchange, decreased time for handovers, as well as increased satisfaction from providers and a perception of improved communication and teamwork with the use of structured handoffs (10).

While simply adopting existing protocols and standardized tools from other centers into practice may seem like a straightforward process, the field of implementation science suggests that in order for an intervention to be successful, attention to implementation strategies are just as important as the intervention itself on exacting change. Mainstays of implementation science strategies and frameworks are identifying local insights, potential barriers, and specifically tailoring the intervention and implementation strategy to the local setting while simultaneously removing barriers (8, 12, 13). As a result, simply adopting an existing handoff protocol may be insufficient at sustaining impactful change, especially when implementing complex healthcare interventions.

This project represents the first stage in a series of quality improvement initiatives aimed at developing and implementing a standardized handoff protocol between the OR and the PCCU, inclusive of structured tools, utilizing quality improvement and implementation science methodology. The first stage required formal evaluation of the current state of handoff practice, assessment of the local environment and review of stakeholder attitudes and perceptions on the handoff practice. The results from this project will aid in root cause analysis and creating process maps which will be used to inform the subsequent projects.

## Methods

The current project was part of a larger project aimed at improving the quality of patient handoffs from the OR to the PCCU. This project included audits and cross-sectional surveys to better understand baseline OR to PCCU handoff performance and provider satisfaction with the current handoff process. This quality improvement project was reviewed and given Research Ethics Board exemption as a QI project.

This project was conducted in a Canadian academic tertiary care children's hospital which is integrated and interspersed within a large adult academic medical centre. The children's hospital is equipped with a 12-bed PCCU, which admits over 800 patients a year, with an approximately 70:30 ratio of medical and surgical patients. At the time of this project, the handoff process at our institution was unstructured and disorganized. There was no policy in place on how the handoff should be given and anecdotally, the handoffs were characterized by individual conversations, interruptions, and missing team members.

### Project team

A multi-disciplinary working group consisting of physicians, nurses, a QI facilitator, respiratory therapist (RT), advanced practice nurse, research coordinator, and human factors engineer was created. This group included representation from the OR, pediatric anesthesia, pediatric surgery, and PCCU.

### Baseline data collection

In order to understand the current process of handoffs between the OR and the PCCU, the working group agreed upon an assessment of the current state. A multi-pronged approach was utilized to obtain both objective and subjective data to inform future process improvement measures.

The working group developed a standard audit tool utilizing examples from the literature as a baseline (Supplementary Table S1). Other areas of interest were added to the audit tool by members of the working group to further assess and understand our patient population and transfer policy. The tool was developed to assess effectiveness of communication, teamwork, preparedness, as well as overall efficacy of the handoff process. All elements on the audit tool were defined prior to beginning the audits. Definitions of subjective elements were agreed upon by the auditors for consistency. This included “PCCU team ready to receive handover” as defined by a staff/resident/fellow of the PCCU team being present for the handoff and the PCCU nurse prepared to accept the patient. As well as a “well organized handoff” being characterized by all team members present, the majority of anaesthesia and surgical elements mentioned, and team members attentive throughout without any interruptions. The audit tool was based on existing frameworks from the literature and in consultation from the multidisciplinary working group/stakeholders. Staff participating in the handoffs were not aware of the audit tool but were informed of the audits taking place.

Initially, audits were performed by two members of the project team (E.W. & E.V.W.) with constant comparison to ensure consistency in the evaluations. Both auditors have clinical backgrounds and have personally participated in patient handovers. The first two audits were completed by both auditors who debriefed after to ensure consistency in their assessments. Audits were completed in person, with the auditor removed from the handover huddle but discreetly placed to be able to hear and observe the handover. Once consistency was confirmed, future audits were performed by one of the two team members. A total of 50 audits of OR to PCCU handoffs were completed between September 2021 and April 2022. Data was documented on the audit tool and subsequently transposed to a Microsoft Excel database (version 2021).

Members of the working group developed a short survey to assess provider perceptions of the current handoff process (Supplementary Table S2). The survey included Likert scale questions as well as free-text options that allowed respondents to provide their perspective and opinions on their previous six-month experience of OR to PCCU handoffs. To ensure accuracy in responses, each section of the survey contained one reverse worded statement that could be correlated with its counterpart.

Data obtained from the surveys provided subjective data from a wider range of providers involved with OR to PCCU handoffs and allowed for a more comprehensive assessment of the current process. Surveys were developed in the institutional Research Electronic Data Capture (REDCap) program and distributed via email link by managers and division leads between April 2022 and June 2022 (14, 15). Email reminders were provided at two-week intervals. Providers surveyed included nurses from the OR and PCCU, RTs from the OR and PCCU, and all pediatric anesthesiologists, pediatric intensivists, and pediatric surgeons. The surveys were distributed to all staff involved in handoffs. However, due to the volume of OR nurses and that only a selection of OR nurses work paediatric cases, a sub-set of relevant OR nurses received the survey. A separate quick comment survey containing one open-text question was created and linked through a QR code that was posted on the QI boards in the OR and PCCU and allowed providers to give real-time feedback on a specific handoff that they had participated in.

### Data analysis

Descriptive statistics were utilized to report frequencies, percentages, and averages across the audits and both surveys. The analysis of the audit data was descriptive in nature and was used to provide baseline values for future interventions. Due to the limited sample size of each group, survey data was combined to capture perspectives from all healthcare providers involved. Likert scale items were recategorized to dichotomous outcomes: always/often/sometimes and rarely/never for data analysis purposes. Analyses were completed using SAS Software (version 9.4; SAS Inc., Cary, NC).

A qualitative thematic analysis was performed on the free-text comments from both surveys by two authors (J.L. & J.D.). A two-person, constant comparative approach based in grounded theory was utilized and consensus achieved through discussion. Analysis was completed utilizing abductive reasoning, which employs both a deductive and inductive approach (16, 17). Themes had been previously identified by the working group and data was coded in a deductive fashion as they applied to the themes. While an inductive approach was also applied to identify new codes and themes that emerged from the data.

## Results

### Audit data

Fifty audits were completed as part of this project. The majority of audits were from Otolaryngology (30.0%), followed by Neurosurgery (26.0%), Orthopedics (24.0%), General Surgery (18.0%), and Ophthalmology (2.0%). Only three (6.0%) cases were multi-service surgeries. The majority (88.0%) of cases were scheduled as elective PCCU stays. On average, it took 28.2 min [standard deviation (sd) = 19.8] from the pre-handoff phone call to arrival in the PCCU. It took an average of 3.1 min (sd = 1.6) from when the patient arrived in the PCCU to the start of the handoff. Finally, the average length of the handoffs was 4.2 min (sd = 1.7).

Overall, the pre-handoff call from the OR to PCCU consistently lacked important elements pertaining to the patient. Intraoperative blood loss (0.0%), precautions (10.0%), and current infusions and rates (0.0%) were consistently missed during these calls (Table 1). Other pertinent information, such as the procedure (82.0%) and airway status (78.0%) were consistently mentioned prior to the patient arriving from the OR. When applicable, technical aspects of the handoff such as the PCCU room being ready (88.0%), ventilator set-up (85.7%) and personal protective equipment being available (75.0%) were consistently completed and available at the time of admission.

**Table 1:** Audit data of handoff elements included in the pre-handoff phone call.

Variables	Audits (*N* = 50)
	% (*n*)
Surgical procedure mentioned	82.0 (41)
Airway (e.g., intubated post-surgery)	78.0 (39)
Intraoperative blood loss	0.0 (0)
Patient precautions	10.0 (5)
Current Infusions & rates	0.0 (0)
Technical items in Paediatric Critical Care Unit (PCCU)	
Room ready for admission	88.0 (44)
Ventilator set up (*n* = 21)	85.7 (18)
Inotropic infusions ready on pump (*n* = 1)	0.0 (0)
Personal Protective Equipment (PPE) ready and available (*n* = 8)	75.0 (6)

Descriptive statistics were based on a sample size of 50 unless otherwise specified.

During the handoff, a representative from each involved specialty was present at 71.4% of handoffs, however introductions were only completed 14.0% of the time (Table 2). Overall, the majority of the anaesthesia content was discussed consistently, with the need for pressors (18.0%) and relevant intraoperative labs/gases (34.0%) as the only elements discussed less than 50% of the time (remaining elements discussed >60% of the time). In contrast, of the 13 elements that were determined to be essential for the surgical team to discuss during the handoff, 7 of the elements were discussed less than 50% of the time. The primary procedure being the element that was mentioned most consistently (78.0%) and the remaining elements being discussed between 13.2%–66.0% of the time.

**Table 2:** Audit data of handoff elements included in the in-person handoff.

Variables	Audits (*N* = 50)
	% (*n*)
Introductions	
Introductions complete	14.0 (7)
Team members present (*n* = 49)	71.4 (35)
Anaesthesiology Information	
Patient identification	80.0 (40)
Relevant medical history	78.0 (39)
Airway discussed	94.0 (47)
Vascular access	86.0 (43)
Intraoperative events or concerns	72.0 (36)
Intraoperative medications given	98.0 (49)
Need for vasopressors	18.0 (9)
Fluids ins/outs	84.0 (42)
Blood loss	62.0 (31)
Relevant intraoperative labs, gases	34.0 (17)
Postoperative analgesia plan	64.0 (32)
Surgery Information	
Procedure performed	78.0 (39)
Brief history of presenting illness/indications for surgery	58.0 (29)
Intraoperative findings (*n* = 49)	65.3 (32)
Intraoperative complications (*n* = 49)	55.1 (27)
Drains/tubes present and plan for care including parameters	46.0 (23)
Surgical site infection prophylaxis	48.0 (24)
Wound care/ostomy care/dressing plan	46.0 (23)
Feeding plan (*n* = 49)	34.7 (17)
Postoperative investigations & timing	38.0 (19)
Potential need for second surgery & timing (*n* = 38)	13.2 (5)
Anticipated postoperative complication	54.0 (27)
Antibiotics duration	66.0 (33)
Parents/guardians updated	28.0 (14)
Operating Room Nurses Information	
Any information not previously mentioned	44.0 (22)
Any additional intraoperative concerns	52.0 (26)
Wrap-up Information	
Summary & plan by PCCU designate	2.0 (1)
Opportunity for questions & clarification	48.0 (24)
Process Information	
PCCU team ready to receive handover	74.0 (37)
No interruptions	36.0 (18)
Number of interruptions	
1	12.0 (6)
2	12.0 (6)
3	8.0 (4)
4	4.0 (2)
5+	2.0 (1)
Opportunities for questions	48.0 (24)
Well organized	32.0 (16)
Closed-loop communication utilized	10.0 (5)
Team members attentive throughout	86.0 (43)
Conducted after patient set up in room	18.0 (9)

Descriptive statistics were based on a sample size of 50 unless otherwise specified.

There was rarely a summary/wrap-up mentioned (2.0%) and only half (48.0%) of handoffs had opportunities for questions and clarification (Table 2). In terms of the handover process, the PCCU team was ready to receive the handover 74.0% of the time. The majority (64.0%) of handoffs experienced interruptions, most commonly between 1 and 2 interruptions. Only 32.0% of handoffs were well organized based on the opinions of the reviewers, with most (82.0%) handoffs occurring before the patient was set-up in the room. In only 14.0% of handoffs did the reviewers believe that team members were inattentive during the handoff. However, in 42.0% of handoffs, the auditors made separate comments describing siloed communication occurring where multiple conversations were occurring simultaneously.

### Provider survey data

Sixty-two healthcare providers completed the survey, representing a response rate of 30.8%. Respondents including 16 (16.7%) PCCU nurses, 6 (100.0%) PCCU staff, 8 (53.3%) Anaesthetists, 7 (35.0%) Surgeons, 18 (60.0%) OR nurses, and 7 (20.6%) RTs. The open-text response from the quick comment survey was also included in this analysis (*n* = 2).

Most staff (60.0%) always/often/sometimes felt unsatisfied with the handoff process, with 18.0% indicating technical errors occurred during the handoff and 22.0% acknowledging that information was missed (Table 3). Possible contributors to the dissatisfaction around the handoff could be related to 36.7% of staff indicating that they always/often/sometimes felt they received insufficient information about the patient. Additionally, over half (67.3%) of staff always/often/sometimes agreed that team members were absent during the handoff and further, 45.3% always/often/sometimes agreed that team members were inattentive.

**Table 3:** Summary of responses from the operating room to paediatric critical care unit handoff provider survey.

Variables	Total responses (*N* = 53)	
	Always/often/sometimes	Rarely/never
	% (*n*)	
Provider-specific Items		
Did not notify/was not notified of direct admit from operating room	13.7 (7)	86.3 (44)
Insufficient information about patient	36.7 (18)	62.3 (31)
Insufficient time to receive patient	23.5 (12)	76.5 (39)
Team members were absent at handover	67.3 (35)	32.7 (17)
Team members were inattentive	45.3 (24)	54.7 (29)
General Survey Variables		
Start and end of handover was unclear	43.8 (21)	56.2 (27)
Anaesthesia report was unclear	16.7 (8)	83.3 (40)
Surgical report was unclear	31.3 (15)	68.8 (33)
Participants/Providers interrupted each other	36.7 (18)	63.3 (31)
Lack of opportunity to wrap-up/clarification	37.5 (18)	62.5 (30)
Unsatisfied with handover process	60.0 (30)	40.0 (20)

Not all survey responses sum to 53, as each survey question was optional and certain respondents may have chosen not to provide an answer.

### Thematic analysis of open-ended survey questions

There were three main themes from thematically analyzing the open-ended survey questions: *missed information, siloed communication, and workflow/timing issues.* Regarding missing information around the handoff, staff expressed how the lack of patient information prior to receiving the patient in the PCCU caused them to be unprepared for the patient. A PCCU staff stated: “*the OR sometimes does not paint an accurate picture of what the patient status is”*. This lack of information not only occurred between departments, but also between staff working in the same setting, with a staff expressing that: “*sometimes the [PCCU] charge RN forgets to give information to the [PCCU] RT about the patient coming from the OR”*.

Staff often cited that this missed information could stem from the frequency of siloed communication that would occur between teams: “*the group comes from the OR, there is no formal pause..nursing talks to nursing, doctor talks to doctors, and I [PCCU nurse] try to listen while setting up the patient”.* The lack of a definitive start to the handoff may be one of the main contributors to this siloed communication, as one of the staff suggested that “*if the nurses are already handing over without anyone else knowing, there are chances for interruptions when the doctors start [handoff]”.*

For these high acuity cases, efficient workflow and timing are essential. Our analysis showed that these cases can be difficult to plan for as there are environmental and patient-specific factors that make the handoff logistically complex. Staff highlighted these issues from personal experience “*depending on time of day, attending PCCU physician may not be present, or may be busy with other patient rounds. Sometimes the attending surgeon is not present, maybe talking to family”.* A staff member provided an example of how a different workflow was effective in other settings of the hospital, suggesting it could be adapted for the PCCU setting, “*In Critical Care Trauma Centre, usually the charge nurse is notified and gives ample notice to RTs”*.

## Discussion

When assessing teamwork, communication, and coordination of complex systems involving multiple individuals, disciplines, and specialties, it is not only important to take a multi-disciplinary approach but also consider a mixed-methods data collection tactic. Our study was unique in that it demonstrated the utility of applying both objective and subjective data collection methods to develop a comprehensive understanding of the current state of OR to PCCU handoffs at our institution.

Both the audit and survey data confirmed that current handoffs were commonly completed without the full team being present and/or had members who were inattentive or distracted through the course of the handoff. The absence of team members during unstructured handoff has been cited as a common occurrence, with the results from the current project falling close to the 60%–70% attendance rates reported in the literature (3, 4, 9). It was also identified in both data collection methods that 40%–50% of handoffs lacked the opportunity for questions and clarification. These findings may be related to the lack of a structured start and end to the handoff, along with evidence of siloed communication where multiple separate conversations occurred simultaneously between different members of the team. The opportunity for questions and clarification is a necessary component of closed loop communication which has been noted to be an effective tool in ensuring information transmission from one team to the next (18, 19). The literature has shown a substantial increase in provider attendance, attention, and invited questions once a structured protocol was implemented for patient handoffs (2–4, 9).

One of the main contributors to the poor handoff performance may come from the fact that introductions were almost never completed at the beginning of the handoff. Despite the fact that some staff do introduce themselves, the decreased importance placed on introductions may stem from the hierarchy within medicine, where staff lower on the hierarchy may feel submissive and that introducing themselves is less important (20). This hierarchy can be dangerous, as studies have shown that an asymmetric status structure creates hierarchies that can lead to poor communication and threats to patient safety (21, 22). Breaking this hierarchy by performing introductions and referring to colleagues by their name can lead to improved communication, prevention of errors, and promote open dialogue (23).

While the different methods of data collection revealed similar findings in certain areas, there were aspects and issues that were only identified with one method or the other. Survey respondents felt that only 20% of handoffs had information missing but review of the audit data suggested a different picture, especially within the surgical handover where the majority of items were discussed less than 50% of the time. The literature supports the fact that the number of technical errors and missing information is higher in unstructured handoffs and has exhibited between a 40%–70% decrease in these incidences once a protocol has been implemented (2, 3, 10). A similar discrepancy was identified in the subjective assessment of interruptions between team members where survey respondents felt this occurred in roughly one-third of cases, whereas audit data suggests that interruptions occur in two-thirds of cases or twice as often as survey respondents thought. Interruptions in healthcare settings are a well-documented issue whereby some interruptions are necessary to alert providers of patient safety concerns (e.g., alarms from patient monitors suggesting abnormal vital signs) while others may be unnecessary and ultimately distract providers' attention away from their primary tasks (24). In our setting, interruptions such as other provider conversations or phone calls to the unit may significantly distract handoff team members from accurately providing a full description of important patient handoff information or for receiving members to fully comprehend messages and tasks.

Other aspects that were only identified on the audits as areas of concern, that have been supported in the literature, included a lack of closed loop communication, the majority of handoffs and communication beginning before the patient was settled with a set of stable vitals, and infrequent opportunities for questions, clarification, and wrap up at the end (9, 18, 19). These findings led our auditors to rate most handoffs as poorly organized. These themes were not identified or specifically commented on by respondents in the survey. In contrast, concepts that the research team was not initially aware of were brought up within the free-text section of the survey by respondents. These included human resource constraints specifically in relation to the RT team as well as other relevant information necessary to prepare the room for patient transfer such as intracranial pressure monitors. Additionally, the added complexity of dual surgical service procedures on the same patient was highlighted as an area to target for improved workflow. Based on respondent comments, in the case of multiple surgical teams performing procedures, the surgical handover was often left to the final surgical team to provide surgical information for all procedures completed despite not participating in the other procedures or having the specialty background.

Collecting data through various collection methods is not only beneficial in confirming and bolstering the concepts and ideas that were expected to be identified, but also provides the opportunity to gather insight and perspective on themes that may only be apparent to certain stakeholders and only identified upon open ended questioning of these participants. This was most evident in the qualitative thematic analysis completed in an abductive fashion, whereby the inductive component allowed our team to identify aspects, concerns and barriers to the handoff process that we were initially unaware of (16, 17). The practice of multi-modality data collection and subsequent analysis is of utmost importance to teams looking to develop and create standardized multi-disciplinary protocols and policies (8, 25). However, the added benefit of robust, multi-modal data collection is the ability to utilize the data to engage other team members in process improvement practices (26). Objective audit data and free-text comments provided by respondents are powerful tools to help other individuals acknowledge and accept problem areas in complex processes. Furthermore, studies have shown that engaging and empowering staff to raise concerns and participate in process improvements builds a safety culture and ultimately leads to preventing poor quality care (26). Finally, administering the survey also allowed our team to identify individuals who expressed interest in participating in the ultimate goal of redeveloping the handoff process, thereby extending the task force into a larger and more inclusive team.

There are several limitations to this study. Selection bias is likely present in both surveys as well as the audits. The selection bias could have worked in both ways, in that respondents may have had an underlying interest in the results and are more invested in the project or alternatively, workload or burnout may have prevented certain staff from responding. The audits were performed primarily on electively booked cases that were completed during daytime hours. Our workforce limitations of daytime research personnel diminished our ability to audit emergency and after-hours cases. While many aspects of a good handoff would be similar for after-hours cases as for electively booked cases, the underlying complexity, severity and potential instability of emergent OR cases as well as the likely reduced after-hours staff are complicating features that were potentially missed in our audits. Additionally, the presence of the auditor could have triggered a change in behaviour due to the Hawthorne Effect in staff (27), however, we do not believe this was the case as audit performance did not improve from the beginning to end of the audits. Finally, some elements of the audit tool were subjective assessments of performance, which may have varied between the different auditors. However, we feel that by completing initial audits together and that most of the audit questions were dichotomous, both auditors were able to come to an understanding on evaluating these subjective measures similarly.

While the current literature has documented the use of standardized tools and pathways for OR to PCCU handoffs, many institutions have simply adopted these tools and assessed the satisfaction and/or efficacy post-implementation or have only utilized a single modality to assess current state handoffs in their institution (2, 3, 10, 11). We describe a robust, multi-modality protocol utilizing both objective and subjective data as well as qualitative data from surveys to evaluate current state handoff processes. This provided the most complete understanding of the needs and perspectives of all parties involved in the handoff process and enabled the creation of a standardized workflow that is targeted to the specific aspects relevant in our institution. Furthermore, completing the survey pre-protocol creation allowed for the expansion of the working group for greater diversity and inclusion in the protocol development process. Other teams looking to develop similar standardized protocols may choose to adopt this process for a robust and inclusive strategy to develop complex multidisciplinary protocols.

Future steps of this QI project include presenting this multi-modal data to all groups of stakeholders. By distributing this information broadly, we can ensure that all parties are aware of the current state of the handoff process and understand the present pitfalls and potential patient safety issues that may arise if current practices aren't improved. Engaging multi-disciplinary team members will aid in building a culture of safety and a team approach to improving our practices (26, 28). Common QI tools such as root cause analyses and process maps will be developed. Subsequently, multi-disciplinary meetings will be conducted to develop a standardized handoff process to address concepts and themes that were identified in the audit and survey data. Recognition of specific barriers that exist within our local environment must be acknowledged by the team and actively mitigated within the new handoff protocol which will be iteratively tested to confirm usability, appropriateness as well as acceptance and satisfaction of use by the multi-disciplinary team.

## Conclusion

The findings from this project helped to identify multiple areas for process improvements in OR to PCCU handoffs. The combination of both objective and subjective data enhanced the ability to understand the current landscape and allows for future planning of quality improvement efforts around a structured OR-PCCU handoff. While other centres may utilize a similar framework to study their handoff processes, they may find different themes and barriers specific to their surroundings. It is important to identify concepts relevant to each complex process through robust multi-disciplinary and multi-modal data collection methods to ensure newly developed protocols fully target and address the needs of all team members and the local environment. The unique structure of our children's hospital nested within a larger tertiary care adult hospital, make the processes and templates that currently exist in the literature difficult to fully integrate. Therefore, these findings will inform the development of a centre-specific structured OR to PCCU handoff process to ensure effective and safe patient care.

## Data Availability

The original contributions presented in the study are included in the article/Supplementary Material, further inquiries can be directed to the corresponding author.
