# Supplementary material for: Evaluating observed and perceived experiences of operating room to paediatric critical care unit handoffs: an initial assessment to inform quality improvement
Source: Front Pediatr. 2025 Oct 8;13:1644064. doi: 10.3389/fped.2025.1644064 (PMC12540124; doi:10.3389/fped.2025.1644064)
Supplement: Supplementary file 1 [file Datasheet1.pdf]

Handover Provider Survey

Project Title: Standardized OR to PCCU Handoffs

What specialty service do you most identify with?	◯ PCCU<br>◯ Anesthesia<br>◯ OR<br>◯ Surgery
What is your role?	◯ Physician<br>◯ Nurse<br>◯ RT<br>◯ Administrator (Managers)
How many years of experience do you have in your current role?	◯ 0-5<br>◯ 6-10<br>◯ >10

When answering these questions think of all the patient transfers from the OR to PCCU you have participated in over the last 6-months.
Please use those cumulative experiences to answer the questions below.

Consider the following statements about receiving a patient:

	Always	Often	Sometimes	Rarely	Never	Not Sure/Unknown
The OR team did not notify the PCCU team that the patient would be admitted directly from the OR.	○	○	○	○	○	○
The OR team gave insufficient information to the PCCU team in order to prepare for the patient.	○	○	○	○	○	○
The OR team gave insufficient time for the PCCU team members to be ready to receive the patient postoperatively at the bedside.	○	○	○	○	○	○
Some team members were absent at the patient handover.	○	○	○	○	○	○
Team members were inattentive to the patient handover.	○	○	○	○	○	○
The OR team adequately informed the PCCU team in anticipation of receiving the patent.	○	○	○	○	○	○

Please elaborate on why you selected 'Always', 'Often' or 'Sometimes' for the question: The OR team did not notify the PCCU team that the patient would be admitted directly from the OR. __________________________________

Please elaborate on why you selected 'Always', 'Often' or 'Sometimes' for the question: The OR team gave insufficient information to the PCCU team in order to prepare for the patient. __________________________________

Please elaborate on why you selected 'Always', 'Often' or 'Sometimes' for the question: The OR team gave insufficient time for the PCCU team members to be ready to receive the patient postoperatively at the bedside. __________________________________

Please elaborate on why you selected 'Always', 'Often' or 'Sometimes' for the question: Some team members were absent at the patient handover. __________________________________

Please elaborate on why you selected 'Always', 'Often' or 'Sometimes' for the question: Team members were inattentive to the patient handover. __________________________________

Please elaborate on why you selected 'Never', 'Rarely' or 'Sometimes' for the question: The OR team adequately informed the PCCU team in anticipation of receiving the patent. ____

When answering these questions think of all the patient transfers from the OR to PCCU you have participated in over the last 6-months.
Please use those cumulative experiences to answer the questions below.

Consider the following statements about receiving a patient:

	Always	Often	Sometimes	Rarely	Never	Not Sure/Unknown
The PCCU team was unaware that the patient would be admitted from the OR.	○	○	○	○	○	○
The PCCU team felt that the OR team gave insufficient information in order to prepare for the patient.	○	○	○	○	○	○
The PCCU team was given insufficient time to be ready to receive the patient postoperatively at the bedside.	○	○	○	○	○	○
Some team members were absent at the patient handover.	○	○	○	○	○	○
Team members were inattentive to the patient handover.	○	○	○	○	○	○
The PCCU team received adequate information from the OR team in anticipation of receiving the patient.	○	○	○	○	○	○

Please elaborate on why you selected 'Always', 'Often' or 'Sometimes' for the question: The PCCU team was unaware that the patient would be admitted from the OR. __________________________________

Please elaborate on why you selected 'Always', 'Often' or 'Sometimes' for the question: The PCCU team felt that the OR team gave insufficient information in order to prepare for the patient. __________________________________

Please elaborate on why you selected 'Always', 'Often' or 'Sometimes' for the question: The PCCU team was given insufficient time to be ready to receive the patient postoperatively at the bedside. __________________________________

Please elaborate on why you selected 'Always', 'Often' or 'Sometimes' for the question: Some team members were absent at the patient handover. __________________________________

Please elaborate on why you selected 'Always', 'Often' or 'Sometimes' for the question: Team members were inattentive to the patient handover. __________________________________

Please elaborate on why you selected 'Never', 'Rarely' or 'Sometimes' for the question: The PCCU team received adequate information from the OR team in anticipation of receiving the patient. ____

Consider the following statements about communication in the handover:

	Always	Often	Sometimes	Rarely	Never	Not Sure/Unknown
The start and end of handover was unclear.	◯	◯	◯	◯	◯	◯
The Anesthesia report was unclear.	◯	◯	◯	◯	◯	◯
The Surgical report was unclear.	◯	◯	◯	◯	◯	◯
Participants/providers interrupted each other.	◯	◯	◯	◯	◯	◯
There was a lack of opportunity for wrap up and clarification.	◯	◯	◯	◯	◯	◯
The handover had a definitive beginning and finish	◯	◯	◯	◯	◯	◯

Please elaborate on why you selected 'Always', 'Often' or 'Sometimes' for the question: The start and end of handover was unclear.

____

Please elaborate on why you selected 'Always', 'Often' or 'Sometimes' for the question: The Anesthesia report was unclear.

____

Please elaborate on why you selected 'Always', 'Often' or 'Sometimes' for the question: The Surgical report was unclear.

____

Please elaborate on why you selected 'Always', 'Often' or 'Sometimes' for the question: Participants/providers interrupted each other.

____

Please elaborate on why you selected 'Always', 'Often' or 'Sometimes' for the question: There was a lack of opportunity for wrap up and clarification.

____

Technical errors were encountered during handover? (e.g., ventilator not available for intubated patient, equipment issues [medications, infusions, PPE])

◯ Yes
◯ No

If yes, please explain

____

Information was missed? (e.g., patient was not identified, past medical history, name of procedure etc.)

◯ Yes
◯ No

If yes, please explain

____

Overall, I am unsatisfied with the current handover process.

◯ Strongly Agree ◯ Agree ◯ Neutral ◯ Disagree ◯ Strongly Disagree

Do you have any other comments or suggestions as to how we can make this process better

____
