# Supplementary material for: Evaluating observed and perceived experiences of operating room to paediatric critical care unit handoffs: an initial assessment to inform quality improvement
Source: Front Pediatr. 2025 Oct 8;13:1644064. doi: 10.3389/fped.2025.1644064 (PMC12540124; doi:10.3389/fped.2025.1644064)
Supplement: Supplementary file 2 [file Table1.docx]

**Supplemental Table 1:** Operating Room to Paediatric Critical Care Unit Handoff Audit Tool

| **Study ID: Procedure: Elective Stay:**  **Date: MRP service: ASA:**  **Observer: PRISM score:** | |
| --- | --- |
| **PRE-HANDOVER** | |
| **Report Called from OR to PCCU:** Time (24hr): ­­­­______________  Procedure: ­­­­­­­­­______________  Relevant PMHx  Airway – *Intubated postop? ­­­*___ *(Y/N)*  Intraoperative blood loss  Precautions  Elective PCCU stay postop? ___ *(Y/N)*  Current Infusions & rates  Any other pertinent information | **Technical items in PCCU**  Room ready for admission (monitor/leads, suction, transfer board)  Ventilator set up (if needed)  Inotropic infusions ready on pump (if needed)  PPE ready and available (if needed) |
| **PCCU HANDOVER** | |
| **1. Introductions**:  Introductions complete  Team members present:  PCCU Staff/Fellow \| Anaesthesiologist/fellow/resident Surgeon/fellow/resident \| OR RN \| PCCU RN \| PCCU RT PCCU Charge RN \| Parents \| Other members:_____________  **2. Anaesthesiologist**  Patient identification  Relevant PMHx  Airway discussed  Vascular access  Intraoperative events or concerns  Intraoperative medications given *(Abx, last paralytic, opioids)*  Need for pressors *(Intraoperative, ongoing)*  Fluids ins/outs *(crystalloid, blood products, urine output)*  Blood loss *(discussed by either anaesthesia or surgery)*  Relevant intraoperative labs, gases  Postoperative analgesia plan  **3. Surgeon**  Procedure performed: ­­­­­­­­­______________  Brief History of Presenting Illness/Indications for OR  Intraoperative Findings  Intraoperative complications  Drains/tubes present and plan for care including parameters  SSI Prophylaxis  Wound care/ostomy care/dressing plan  Feeding plan  Postoperative investigations & timing (*labs, imaging*)  Potential need for second surgery & timing  Anticipated postoperative complication  Antibiotics duration (if needed)  Parents/ guardians updated | **4. OR RN**  Any info not previously mentioned (*PMHx, allergies etc.)*  Any additional intraoperative concerns  **5. Wrap-up**  Summary & plan by PCCU designate  Opportunity for questions & clarification  **6. Process**  PCCU team ready to receive handover  No interruptions _________ (*number)*  Opportunities for questions  Well organized  Closed-loop communication utilized  Team members attentive throughout  Conducted after patient set up in room |
|  | **COMMENTS** |
|  | Patient Arrival (24hr): ______________  Handover Start (24hr): ______________  Handover Stop (24hr): ­­­­______________ |
